# Development, Implementation, and Evaluation of a New Course on Essential Skills for Women’s Leadership in Global Health

**DOI:** 10.5334/aogh.3730

**Published:** 2022-07-11

**Authors:** Anna Kalbarczyk, Elizabeth Hood, Luthfi Azizatunnisa, Utsamani Cintyamena, Frehiwot Nigatu, Prativa Baral

**Affiliations:** 1 Center for Global Health, Johns Hopkins University, Department of International Health, Johns Hopkins Bloomberg School of Public Health, US; 2 The Ann and Robert H. Lurie Children’s Hospital of Chicago, School of Nursing, Johns Hopkins University, US; 3 Department of Health Behavior, Environment and Social Medicine, Faculty of Medicine, Public Health and Nursing, Universitas Gadjah Mada, ID; 4 Center for Tropical Medicine, Universitas Gadjah Mada, ID; 5 International Institute for Primary Health Care-Ethiopia, ET; 6 Department of International Health, Johns Hopkins Bloomberg School of Public Health, US

**Keywords:** course, training, women’s leadership, essential skills, evaluation, global health

## Abstract

While many calls have been made to support the development of women leaders in global health, few resources have been developed and evaluated to meet this goal. We developed and evaluated a one week online short course on the essential skills for women’s leadership in global health, offered in June 2021 to 22 students from 4 countries (Australia, Ethiopia, Thailand, and the United States). The course covered the state of women’s leadership in global health and influencing factors; leadership theories models and frameworks; self-awareness and self-assessments; organizations and enabling environments; communication; and negotiation, and was designed to promote skills via practice, discussion, and debrief. Students rated the course highly and enjoyed the skills-building components, diversity of voices presented throughout the course, and embedded networking opportunities. Future iterations of the course, particularly those held in low-and middle-income countries, should contextualize materials, co-create with local instructors and amplify local voices, and consider incorporating shadowing, coaching, mentorship, and communities of practice.

## Introduction

While many calls have been made to support the development of women leaders in global health [1,2,3], and to promote intersectional approaches [4], few resources have been developed, documented, and evaluated to meet this goal [5].

Savvy leadership in global health is of great importance now more than ever as we widely grapple with the colonial and patriarchal underpinnings of current global health practice and research [6]. Men continue to hold the majority of leadership roles in global health, while 70% of the global health workforce are women [3]. And in institutes of higher learning, public health (and global health) students continue to be overwhelmingly women. But women recent graduates, in comparison to their peers who are men, are not receiving formal leadership training [7]. Women’s limited representation in leadership is compounded by the intersection with other factors including race, religion, caste, class, and ethnicity [3]; women leaders of different races are perceived differently, facing racial and gendered biases simultaneously [8].

As women continue to grow in the global health workforce, we must be better prepared to strengthen their leadership skills in preparation for cadres of women leaders who will propel the field forward.

## Formation of a women’s leadership in global health course

In 2020, the Johns Hopkins Center for Global Health (CGH) launched a seminar series to explore the lessons learned from women leaders in global health. One clear theme that emerged across these talks was a call for more skills-building for emerging women leaders, particularly in essential skills [9]. This call catalyzed the development of a 3-credit course on “Essential Skills for Women’s Leadership in Global Health” that was first offered at the Johns Hopkins Bloomberg School of Public Health, during its summer institute, in June 2021. This Institute is historically offered only in-person, but the ongoing COVID-19 pandemic provided an opportunity to develop the course for a synchronous online audience (including students from Australia, Thailand, and Ethiopia, who might otherwise not have been able to join).

The development of the course was broadly guided by our speakers’ lessons learned in leadership. These lessons were collated and key themes were extracted to identify critical areas for learning and development, including following a passion, not a plan; building bridges, not dams; and strengthening leadership skills [9]. Existing leadership competencies for global and public health, where available, were reviewed to cross-check curriculum for critical gaps but were not explicitly used to guide the course development. The goal throughout course development was to establish a course that transformed the lessons learned from our seminar speakers into skills-oriented materials and exercises that could promote the growth of leadership skills for women in global health.

## Course content

The course was designed to limit the amount of time spent on lectures and maximize active participation, skills-building, and reflection; all lectures were accompanied by at least one activity. Efforts were made to diversify the range of voices included in the course—in addition to lectures by core teaching faculty, students were asked to listen to podcasts and webinars given by a diverse and global set of speakers. The final day featured a panel of women leaders from Nigeria and India with experience across Africa and Asia.

The content domains covered in the course included an introduction to the state of women’s leadership in global health; leadership theories, models, and frameworks; self-awareness and implicit biases; communication (about oneself, one’s work, and with others in a team); and negotiation. Table 1 shows the domains, topics covered, and associated activities.

**Table 1:** Course domains, specific topics, and related activities.


DOMAIN	DIDACTIC TOPICS	RELATED ACTIVITIES

State of GH Leadership and documented barriers for women leaders	Women’s representation in leadership Priority setting in global health Glass Cliff Intersectionality Structural inequities Gender bias and discrimination Sexual harassment “Balance” Networks and Mentorship	Discussion Case Study—the Glass Cliff Final day panel of speakers

Leadership Theories, Models, and Frameworks	Historical leadership theories/styles (i.e., trait, behavioral, contemporary) Gender-inclusive frameworks Cross-cultural leadership Authentic leadership Inclusive leadership	Polls and discussion Case Study—Inclusive leadership

Organizations and Enabling Environments	Organizational culture and climate Segregation and stereotyping Queen Bee phenomenon Maternal Wall	Identifying leadership values Group leadership SWOT

Self-Awareness	Self-assessments Implicit Biases—“Traits” of Women Leaders Imposter syndrome Freedom to Fail	Pre-course self-assessment Case study—inclusivity Discussion—share a failure and lessons learned Final assignment

Communication	Gender and communication Communicating about ourselves Developing teams through communication Active Listening Coaching Science Communications	Elevator pitch Active listening Presentation

Negotiation	Benefits of negotiation Approaches to negotiation	Discussion—experiences with negotiation Case study, practice, and debrief


Prior to the first day of the course, students were asked to complete one or more self-assessment tools and reflect on their results, either individually or with members of their personal and professional networks. We selected both free and paid tools that focused on leadership skills [10], authenticity [11], and emotional intelligence [12] and could prompt students to reflect on their strengths and weaknesses. This self-reflection formed the basis of many subsequent conversations and set a tone for ongoing reflection and awareness.

### State of women’s leadership in global health

We began the course with a discussion on the current state of women’s representation in leadership globally and how that affects priority setting in global health. Students were introduced to the concept of the glass cliff—where women tend to be promoted to leadership roles during times of crisis when chance of failure is more likely. Other well-documented barriers to women’s leadership including structural inequities, gender bias and discrimination, sexual harassment, the myth of balance, and reduced access to networks and mentors were also described and discussed [13].

### Leadership theories, models, and frameworks

With this background, we explored leadership theories, models, and frameworks and their applications to women’s leadership specifically. Modern theories including authentic leadership and inclusive leadership were discussed in-depth, including drawbacks and critiques of these theories as applied to women in global contexts. Because of the course’s focus on global health, we included content on cross-cultural leadership, and recognized that leadership approaches don’t work well in all contexts and for all types of organizations.

Leadership does not occur in a vacuum, so we believed it was critically important to discuss the role of organizations and enabling environments in supporting (or hindering) women’s leadership globally. We differentiated between organizational culture and climate and discussed examples of organizational segregation and stereotyping (i.e., gender, racial, ethnic). We also introduced two phenomena that affect women’s leadership and careers: the maternal wall—the discrimination mothers face in their jobs and in searching for employment, and the queen bee phenomenon, a derogatory term applied to women leaders who are seen to distance themselves from junior women [14].

### Building essential skills

We then tackled three specific ‘essential skills’ for leadership including self-awareness, communication, and negotiation.

1. Self-awareness—In addition to the initial self-reflection assignment, we also spent time in class discussing implicit and explicit biases related to gender and race and the role of awareness in reducing women’s susceptibility to negative effects. Students described personal experience with imposter syndrome (feelings of self-doubt despite evidence of the contrary). We further explored how people rarely have an accurate assessment of their own skills and capability, falling somewhere on a continuum between overconfidence and uncertainty. We also explored biases within self-assessments describing their fallibilities, proposing solutions, and discussing the importance of continuous self-reflection as individuals change over time.
  This domain also explored ‘failure’ as a concept related to leadership growth. We shared stories of our failures, the pivotal lessons learned, and the simultaneous fear associated with failures (in spite of their immense benefits).
2. Communication—Communication was divided into three sections: a) communication about yourself to others, b) team communication (i.e., active listening), and c) communicating as an expert about a specific topic. Language and communications play a role in how we understand gender; gender roles are evolving and changing but traditional roles still persist in communication styles.
  To practice communicating about themselves, students were asked to prepare a short elevator pitch introducing themselves by defining the purpose of their pitch (the why) and describing their value proposition or uniqueness. We encouraged students to use a storytelling approach to align their goals with the goals of their target audience. We also spent time discussing the value of networks and how increased contact, effective communications, and strong relationships can increase the likelihood of being remembered or considered for leadership roles.
  We then explored team building through communications and using communication tools to effectively manage and maneuver diverse teams. Here we focused on active listening, “listening with the goal to develop a clear understanding of the speaker’s concern and to clearly communicate the listener’s interest in the speaker’s message [15].” We discussed barriers to active listening (i.e., presence, perceptions, and verbal communications) and strategies to overcome these barriers. Students were then placed into groups of three and worked together, through active listening, to set a leadership agenda or identify a problem statement. The goal was not to find a solution, but rather to arrive at a better understanding of a core problem.
  Given the ongoing COVID-19 pandemic and onslaught of misinformation we felt it was important to build skills in science communication, considering gender and sociocultural barriers and stereotypes. We discussed power dynamics in global health, the type of knowledge that is valued [16], and how we might elevate the voices of women, BIPOC, non-binary, trans, and marginalized communities within global health. In small groups of three, students were asked to present something related to their current scientific work; teammates then asked questions, using an interview-style approach.
3. Negotiation—We described the benefits of negotiation as a leadership skill in terms of team management, collaboration generation, and salary, and presented core concepts in negotiation (i.e., anchoring). Students were given a case study and assigned specific roles and perspectives (i.e., job applicant or hiring manager). In groups of two, students then negotiated a starting salary and other terms of employment. We collated results from each team, discussed the process, and future negotiation strategies.

Throughout the course, discussion reigned supreme. We used polls, guiding questions, virtual white boards, breakout rooms, and plenary debriefs to give students a range of mechanisms for participation and sharing.

## Course Evaluation

The Johns Hopkins School of Public Health sent standard end-of-course surveys to students immediately after the course ended in June. Each student has two weeks to respond, and the data is aggregated to maintain confidentiality. Of the 21 students enrolled in the course, 14 responded to the school’s survey, a response rate of 67%.

The course received excellent ratings overall. The course organization was rated as excellent (n = 11, 78.6%) or good (n = 3, 21.4%). The class assessments (tests, papers, etc.) were rated as excellent (n = 10, 71.4%) or good (n = 4, 28.6%). The other item evaluated through this traditional method included that the course expanded knowledge related to the topic and improved skills (excellent, n = 11, 78.6%, good, n = 3, 21.4%). The respondents also rated the instructor and teaching assistant highly.

We designed a qualitative follow-up survey via Google Forms to obtain more feedback from students about their experience with the course and its content. This survey included six open-ended questions (included in Table 2), was designed to take less than 15 minutes to complete and was circulated six months after the course, in December. This study was approved by the Johns Hopkins Bloomberg School of Public Health Institutional Review Board. Twelve students (57%) responded to this survey.

**Table 2:** Qualitative Survey Questions.


QUESTION NUMBER	QUESTION

1.	Please tell us where you are in your career, and how this course was applicable to this stage of your career.

2.	What elements of the course surprised you and your understanding of leadership, and yourself?

3.	Overall, what did you get out of the course?

4.	What else might you have needed from the course, in terms of skills, ability to implement the material, and any other forms of application?

5.	What are ways in which you are envisioning applying the content you have learned in the course?

6.	What recommendations do you have for us if we were to offer this course in other settings and contexts (in-country and globally)? Specifically, in an international context (outside of the USA), which elements of the course would be applicable; which ones do you think would not be applicable?


We reviewed the data using a qualitative content analysis approach [17]. Responses were analyzed into meaning units, condensed meaning units, codes and then finally themes. The themes were grouped to reflect the number and types of comments. As the themes were reviewed, a number of strong thematic elements emerged across all of the questions.

Unsurprisingly, the most common theme was related to leadership skills, competency, and the use of introspection to better understand the student’s innate leadership style. The class enjoyed learning new **leadership skills and understanding how their own leadership has an impact on their relationships at work**.

*I’m in senior leadership, and the class asked me to re-examine leadership styles in context of gender--it was really helpful in thinking through how much I have been socialized to behave in certain ways*.

*I have 3 years of experience, and was thankful to have this insight at the beginning of my career. I immediately tried negotiating my salary after the class ended, and am excited to see the impact play out in the rest of my career*.

*Just being aware of the glass cliff has helped me more critically reflect on opportunities that I have been presented and those that I see other women tackling*.

Another common theme **involved relationships with more senior women**; the class identified a need for mentoring in their own careers and in the context of negotiations. Networking and interacting with other people during the course was valuable to participants. They found the course content around negotiations, glass ceilings, and maternal cliffs compelling and one student commented that the course was “life-changing.”

*I am in a leadership position in my organization and unfortunately experienced a “maternal wall” situation. This course helped me understand my value and how to better negotiate and navigate as a woman in the field for myself personally, as well as to better mentor those I work with under these same learnings*.

Another important theme was related to **storytelling and learning new skills to share knowledge and information in the workplace**. Learning to tell their own stories from a new perspective and in the context of gender and leadership was meaningful to students.

*The coaching portions were insightful. It was nice to be able to mentor others and I did not realize I had as much as I did to offer, like helping others negotiate, or sharing my story*.

Some students indicated they would like more information about global contexts and international work communities. They had concerns about strong patriarchal societies around the globe and wanted strategies for working within those communities. They also wanted **more time in the course** to explore these complex interpersonal dynamics and would have enjoyed role-playing some scenarios. They also desired additional time with self-reflection, networking and having more access to mentors, formally assigned as part of the course.

*The course was great. I think it would have been nice to have more networking within the group, but it was challenging not being in person to do so*.

After the course was completed, students were asked to consider the ways in which the course would impact their professional lives. Students expressed gratitude for the information and expressed interest in **sharing their new perspectives** with others in their fields and countries.

*I am constantly applying the coaching and communication techniques we learned in my daily life professionally, academically, and personally. The course was also timely in that I learned negotiation skills several days before receiving my new job offer and attempting to negotiate. While I was not able to gain additional compensation due to HR policies, I felt comfortable advocating for myself and asking for more than was offered*.

One student answered that the class had value in demonstrating “that I wasn’t the only one thinking about certain issues, that it is up to ourselves to stand up for us, that sexism prevails in a bigger way than once thought.”

## Future iterations of the course

We have seen growing global interest in promoting women’s leadership in global health, and organizations such as WGH and WomenLift Health have gathered large followings. Our own virtual network for emerging women leaders, established in Slack, has gathered 780+ members in the past year alone. We also know that women leaders in different contexts are facing similar challenges. We have an opportunity to respond to this interest to meet the needs of emerging women leaders globally.

Existing leadership programs in global health focus broadly on the many competencies needed to succeed in global health, from “essential skills” to “technical skills [18,19].” This course recognizes that opportunities for gaining content expertise and technical skills are more widely available (i.e., through degree programs and online training) than leadership training [20]. Long-term global health fellowship programs that incorporate leadership components (i.e., Fogarty Fellowships, Global Health Corps, Sustaining Technical and Analytic Resources [STAR], and Afya Bora) can also require long time commitments and may be less widely accessible for individuals in various stages of their careers. A recent assessment of the STAR training also identified an important skills gap among participants related to gender equity, ethics, and health equity and social justice [20]. This course represents an opportunity to offer a gender equity-oriented leadership curriculum to those who may need it most and in early stages of the leadership journey.

Moving forward, we seek to adapt this course to other settings to serve as a resource for emerging women leaders in global health to gain skills relevant to their career paths. Based on the evaluations, engagement with the literature on best practices, and our own discussions, we propose approaches for future iterations and adaptations.

### Contextualizing material

Implementation science recognizes “context as king,” noting that an intervention’s content *and* its process (i.e., how it is delivered, and by whom), must be tailored to specific contexts to meet local needs [21]. We recognize the essential value of context in course material and propose to develop country-specific examples and case studies, use local/national/regional statistics, and invite local guest speakers to share their stories. We also believe it is important to incorporate anti-oppressive teaching principles in course delivery mechanisms to address power differentials and colonialism [22]. This includes co-creation of leadership competencies, materials, and delivery approaches with in-country partners, interactive and equal participation opportunities regardless of social status, regional origin, and culture, as well as opportunities to lead in the local setting. As students noted, it is important to recognize existing systems and the extent of the patriarchy within those systems so that discussions and recommendations are not tone-def. For example, this could include expanding the traditional meaning of “women leaders” as women who lead “over” men, instead developing context for women as leaders in their own right, in their many roles. Additionally, negotiation materials should be tailored to local negotiation cultures and approaches, currencies, and reasonable salaries.

### Training Instructors and recruiting students

Local instructors and/or facilitators should be involved in the development and delivery of the course (via a training-the-trainer approach [ToT]) given their intimate knowledge of the local context. In a recent Good Health Research Practice (GHRP) training [23], the organizing committee engaged local facilitators not only to provide assistance more easily, but also to bridge with the instructors and other facilitators from different countries with different backgrounds. Involving local instructors builds capacity to lead courses and hold leadership training independently in the future.

ToT approaches have been widely used in global health but with varying success; while ToT is assumed to be self-sustaining, this is not always the case [24]. Identifying the right candidates, providing long-term resources (i.e., funding), and integrated nurturing/development opportunities are needed to sustain ToT models and support new trainers [24]. We propose that future adaptations plan for long-term partnerships, connect instructors and students to existing networks, and offer a mentorship component. Mentorship has been widely lauded as a valuable tool that can support career growth for women in global health [25,26]. We propose incorporating a mentorship model for local instructors and students.

The selection of participants from varied backgrounds can help increase awareness of marginalized voices in this classroom, such as women from remote areas and groups with disabilities. Our students reflected on the value of the diversity of voices and range of experiences represented in the classroom, and that networking was therefore inherently built into the course. We note, however, that the student selection process will likely face biases both because of those making the selection and the smaller percentage of women from remote areas who might hear about the course or be able to attend. Some strategies for inclusive recruitment include partnering with other schools, ministries, local organizations, and community health worker groups to advertise as broadly as possible (and in local languages).

There may also be opportunities for programs to formally embed this course into existing global health curricula. This allows students to take the course for credits that can be applied to their degree or certificate program.

### Modified and additional approaches

Based on course feedback and our subsequent discussions, we also identified approaches to modifying existing curriculum and activities. This includes ensuring students are paired/grouped from different settings to increase knowledge exchange and to create local communities of practice (CoP) that can foster continuous sharing and storytelling. This CoP can be linked with other existing CoPs locally, regionally, and/or globally to grow students’ networks, and as an open opportunity to recruit future instructors for local courses. Given our own access to a virtual Slack networking space, we also discussed providing dedicated linkages for students to this network. For example, students could be provided dedicated Slack channels for networking or discussing course content, within the larger Slack workspace. This approach sensitizes them to the process of engaging virtually, adds Slack to their arsenal of virtual tools, and provides ongoing connection within classmates beyond the scope of the course.

Coaching is an important part of releasing a leader’s full potential and while we discuss the value of coaching in the course, and practice active listening, there is not yet a formal coaching process. Certified coaches could provide coaching to trainees as part of the curriculum, allowing them to connect their day-to-day experience with the training components. This can support goal setting and establish environments in which emerging leaders are solutions-oriented. The benefits are many: students receive direct benefits from coaching that can be applied to their leadership journeys immediately; local trainers can collate lists of local coaches, establishing a cadre of certified go-to coaches; and course participants can spread coaching values within their own workplace and begin to assist others on their teams.

Studies also support the use of shadowing as a pedagogical approach that helps studies build critical competencies including communication, teamwork, respect for other professions, and introducing new roles (i.e., leadership) [27]. Course participants could be connected with local, regional, and/or national women leaders as part of the course and given an opportunity to shadow their day-to-day activities. This would provide learners unique opportunities to observe critical leadership abilities in action and ask questions.

Many of these suggestions, though, including student recruitment, the community of practice, mentorship, coaching, and shadowing, take remarkable time and financial resources to implement. Senior leaders who participate in the program must be incentivized and appropriately compensated for their time and energy. This is particularly salient for women of color, who are overwhelmingly and historically burdened by expectations of “volunteer” labor or service to their institutions [28].

## Conclusion

Resources to advance women leaders in global health are sorely lacking and this course on essential leadership skills provides one training model for institutions to support emerging women leaders. Participants discussed the value of having a diverse set of voices participate in the course and enjoyed the emphasis on discussion, reflection, and skills-building. Future iterations, even if offered in specific countries, should maintain the incorporation of diversity, sharing, and storytelling.
